# Intranodal glue embolization for lymphorrhea following inguinal lymphadenectomy in penile cancer: A case report

**DOI:** 10.1016/j.eucr.2023.102630

**Published:** 2023-11-26

**Authors:** Taisuke Okumura, Shiro Onozawa, Takumasa Amemiya, Yuki Matsumoto, Masayasu Urishibara, Kazuhiro Ishizaka

**Affiliations:** a Department of Urology, Teikyo University Hospital, Mizonokuchi, Kawasaki-shi, Kanagawa, 213-8507, Japan; b Department of Radiology, Faculty of Medicine, Kyorin University, Shinkawa, Mitaka-shi, Tokyo, 181-8611, Japan

**Keywords:** Penile cancer, Inguinal lymphadenectomy, Lymphorrhea, Intranodal glue embolization

## Abstract

An 86-year-old man underwent total penectomy and bilateral inguinal lymphadenectomy (ILND) for penile cancer with an enlarged right inguinal lymph node. The accumulation of 100–150 ml of lymphatic fluid was observed in the right inguinal drain in a day after surgery. Compression was performed, without any improvement in lymphorrhea. During the right inguinal lymphangiography performed on postoperative day (POD) 28, lymphorrhea was still detected. Lymphorrhea was improved 2 days after intranodal glue embolization (IGE) was performed using a mixture of lipiodol and n-butyl-2 cyanoacrylate (NBCA). IGE was effective for intractable lymphorrhea after ILND in penile cancer.

## Highlights

• Lymphorreha after inguinal lymphadenectomy for penile cancer is intractable.
• There is no established management protocol for inguinal lymphorrhea.
• Intranodal glue embolization was effective for lymphorrhea post-lymphadenectomy.

## Abbreviations

CT: computed tomography
IGE: intranodal glue embolization
ILND: inguinal lymphadenectomy
MRI: magnetic resonance imaging
NBCA: n-butyl-2 cyanoacrylate
VAC: vacuum-assisted closure
POD: postoperative day
SCC: squamous cell carcinoma

## 1 Introduction

Complications are common after ILND in penile cancer. One of them, seroma, has an incidence rate of 5%–13.8 %; while another, lymphocele, has an incidence rate of 2.5%–5.2 %.^1^ Inguinal lymphorrhea is intractable with no established management protocol, resulting in a prolonged hospital stay. Recently, intranodal glue embolization (IGE) for inguinal lymphorrhea has been reported as efficient.^2^ Therefore, we aimed to perform IGE for this case of intractable inguinal lymphorrhea following ILND to see the improvement in lymphorrhea. To the best of our knowledge, this is the first report of IGE for inguinal lymphorrhea following ILND in penile cancer.

## 2 Case presentation

**Fig. 1:**
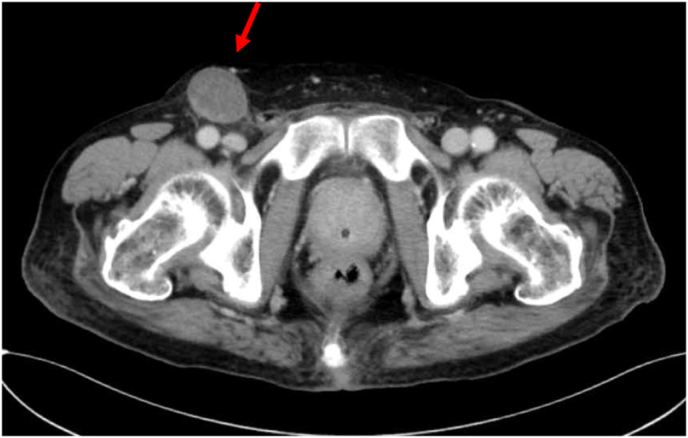
Contrast-enhanced computed tomography revealing an enlarged 3-cm right inguinal metastatic lymph node in penile cancer before lymphadenectomy (arrow).

An 86-year-old Japanese man with true phimosis and a tumor of the penile glans. A lymph node was moveable and palpable in his right groin. Contrast-enhanced computed tomography (CT) and magnetic resonance imaging (MRI) revealed that the tumor invaded the penile glans, and an enlarged lymph node measuring 3 cm was found in the right groin. The patient was diagnosed with penile cancer at a clinical stage of T1N1M0 (Fig. 1). A biopsy of the glans tumor and the enlarged right inguinal lymph node was performed, and histopathology revealed it was a squamous cell carcinoma (SCC). The patient requested for definitive tumor resection; therefore, he uenderwent total penectomy, perinial urethrostomy, and bilateral ILND. Intraoperative pathological diagnosis of the left superficial inguinal lymph node biopsy revealed no metastasis of penile cancer, so limited ILND was performed and standard ILND was performed on the right side. ILND was performed through a skin incision parallel to the inguinal ligament. Lymphatic vessels were resected using a silk thread and vessel seaing device. 10Fr closed suction drains were placed in the bilateral inguinal regions. Histopathology revealed SCC of the penis, pT2, pN1(1/18: right superficial inguinal lymph node metastasis only).

**Fig. 2:**
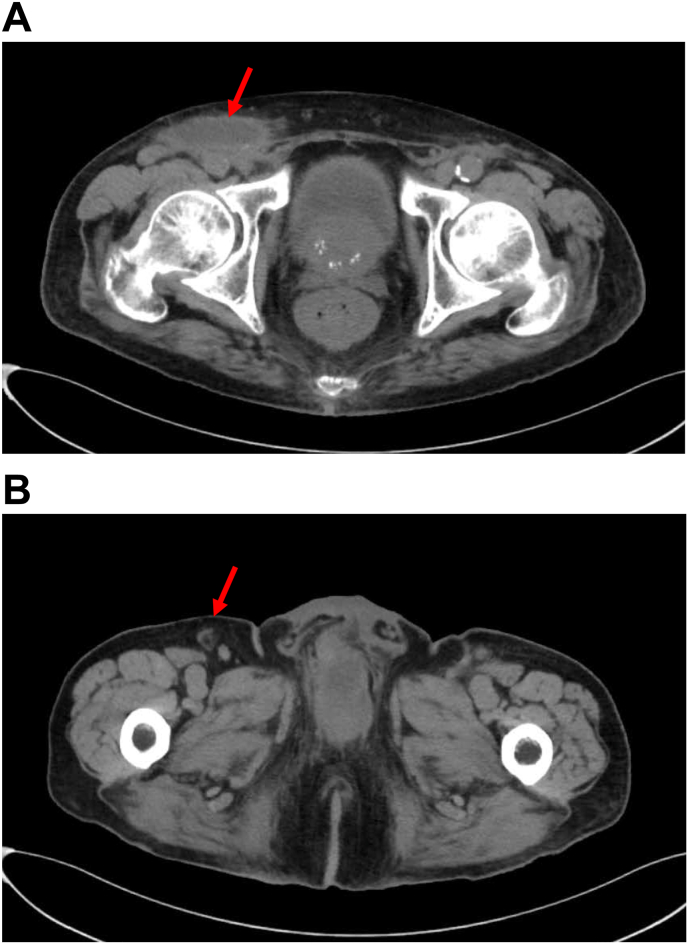
Plain computed tomography taken after right inguinal lymphadenectomy (POD 27) (A) Lymphocele in the right groin (arrow). (B) Residual caudal lymph node measuring 8 mm in the right groin. Lymphangiography was performed via this lymph node (arrow).

After surgery, lymphatic fluid drainage continued flowing at the rate of 50 ml/day through the left inguinal drain. On POD 11, the left-side lymphatic fluid drainage disappeared, so the drain was removed. On average, 100–150 ml of lymphatic fluid accumulated in the right inguinal drain per day. On POD 20, the 10Fr closed suction drain was replaced with a 4 mm Penrose drain, and compression therapy on the right groin was attempted; however, the lymphorrhea flowing from the drain did not decrease. On POD 27, plain CT revealed a right inguinal lymphocele and slightly residual right caudal inguinal lymph nodes (Fig. 2A and B). Therefore, inguinal intranodal lymphangiography was performed on POD 28.

**Fig. 3:**
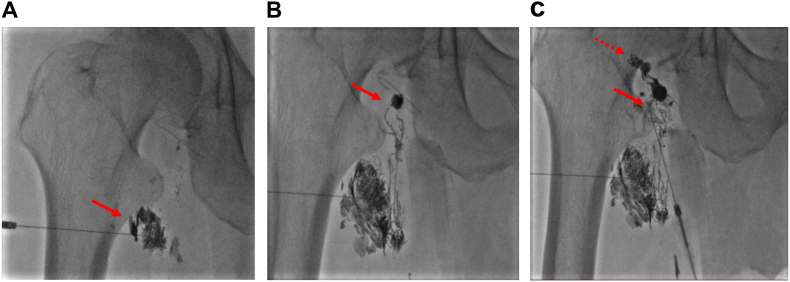
Fluoroscopic image revealing lymphangiography using lipiodol (A) The residual caudal right inguinal lymph node was punctured using a 23 G 60-mm needle under ultrasound guidance and lipiodol was injected (arrow). (B) Intranodal lymphangiography revealing lymphatic leakage from the lymphatic duct (arrow). Intranodal glue embolization was performed using the lipiodol–n-butyl-2 cyanoacrylate (NBCA) mixture. (C) Furthermore, the lymphatic duct was directly punctured under fluoroscopy (arrow). The cranial lymphatic leakage (dashed arrow) was observed and embolized using the lipiodol–NBCA mixture.

Under ultrasound guidance (using a 10 MHz linear probe), an inguinal lymph node was detected and punctured using a 23 G 60-mm needle (Terumo Cattelan Needle, Terumo, Tokyo, Japan). Lymphangiography was performed using ethiodized oil (Lipiodol, Guerbet Japan, Tokyo, Japan). At the first puncture, lymphatic vessels leading from this lymph node to the cranial side were visualized (Fig. 3A), and lymphorrhea was confirmed (Fig. 3B); therefore, embolization was performed using n-butyl-2 cyanoacrylate (NBCA; Histoacryl; B. Braun, Melsungen, Germany) glue mixed with lipiodol in a 1:1 ratio. Furthermore, the lymphatic duct was directly punctured under fluoroscopy and lymphorrhea was confirmed on the cranial side, so embolization was performed (Fig. 3C). The total injection volume of the NBCA and lipiodol mixture was 0.6 ml. As a result, lymphorrhea was confirmed at two sites. On day 2 (30 days after the first surgery) after IGE, lymphorrhea disappeared and the Penrose drain was removed. On day 7 after IGE, the patient was discharged. After 6 months of IGE, CT revealed no recurrence of lymphorrhea or lymphadenopathy, and no complications were observed.

## 3 Discussion

Patients who develop lymphorrhea after ILND for penile cancer have worsened quality of life due to longer hospitalization periods. The percutaneous drainage of lymphatic fluid, compression of the affected area, and sclerotherapy are carried out as conservative treatments for inguinal lymphorrhea after ILND; however, the efficacies are limited. When conservative treatment fails, vacuum-assisted closure (VAC) is useful for inguinal lymphorrhea. Additionally, the median duration of VAC is relatively long (18 days; range: 13–29 days), and continuous device placement is required.^3^ In recent years, inguinal approach lymphangiography for inguinal lymphorrhea has been reported to be efficient. Intranodal lymphangiography with lipiodol through the inguinal lymph nodes can identify lymphorrhea.^2^ In this case, CT revealed residual caudal lymph nodes in the right groin, which was of normal size (Fig. 2B). Lymphatic fluid usually flows from caudal to cranial. Therefore, we believed that this lymph node was not involved in metastasis and performed embolization for inguinal lymphorrhea using the inguinal approach. Per the method proposed by Ozawa et al., the lipiodol–NBCA glue mixture is injected to embolize lymphorrhea. A high success rate (100 %) has been reported for IGE using NBCA for inguinal lymphorrhea. The concentration of NBCA glue for IGE ranged from 16.7 % to 33.3 %, and the total injection volume was reported to be only 1.5 ml in one case. The median number of treatment cycles required to achieve clinical success was two (range: 1–3). The median treatment duration after IGE was 2 days (range: 1–13 days). The most frequent etiology of inguinal lymphorrhea was femoral vessel catheterization (66.7 %). No complication was observed.^4^ We performed IGE with a 50 % Lipiodol–NBCA mixture. A single administration was sufficient for this treatment, the lymphorrhea was improved two days after and there were no complications. IGE is minimally invasive and may shorten the treatment period compared to VAC.

There have been no reports of IGE for inguinal lymphorrhea after ILND for penile cancer, and this is the first of such case reports. Sosogi et al. reported the case of inguinal lymphorrhea after femoral vessel resection, which was unsuccessfully managed through ILND and VAC therapy. However, they were able to successfully perform IGE because of the residual caudal inguinal lymph node.^5^ However, this treatment may not be possible if no inguinal lymph nodes remain following lymphadenectomy.

## 4 Conclusion

Lymphorrhea, a postoperative complication of ILND for penile cancer, is intractable. IGE using NBCA to treat inguinal lymphorrhea is safe and effective and may shorten the patient's hospital stay.

## Ethics in publishing

The requirement for ethical approval was waived by our institution since the case report was retrospective and fully anonymized. There were no ethical concerns regarding this article.

## Informed consent

Written informed consent was obtained from the patient for his anonymized information to be published.

## Funding

The researchers did not receive any specific grant from funding agencies in the public, commercial, or not-for-profit sectors.

## CRediT authorship contribution statement

**Taisuke Okumura:** Conceptualization, Supervision, Writing – original draft, Writing – review & editing. **Shiro Onozawa:** Conceptualization, Resources, Supervision. **Takumasa Amemiya:** Data curation, Investigation. **Yuki Matsumoto:** Data curation, Investigation. **Masayasu Urishibara:** Writing – review & editing. **Kazuhiro Ishizaka:** Conceptualization, Supervision, Writing – original draft.

## Declaration of competing interest

None.
